# Risk for cardiovascular disease associated with metabolic syndrome and its components: a 13-year prospective study in the RIVANA cohort

**DOI:** 10.1186/s12933-020-01166-6

**Published:** 2020-11-22

**Authors:** María J. Guembe, Cesar I. Fernandez-Lazaro, Carmen Sayon-Orea, Estefanía Toledo, Conchi Moreno-Iribas, for the RIVANA Study Investigators, Joaquín Barba Cosials, Jesús Berjón Reyero, Javier Díez Martínez, Paulino González Diego, Ana Ma Grijalba Uche, David Guerrero Setas, Eduardo Martínez Vila, Manuel Serrano Martínez, Isabel Sobejano Tornos, José Javier Viñes Rueda

**Affiliations:** 1 Department of Health, Government of Navarre, Vascular Risk in Navarre Investigation Group, Pamplona, Spain; 2 Dirección General de Salud del Gobierno de Navarra, Servicio de Planificación, Evaluación Y Gestión del Conocimiento, Pamplona, Spain; 3 Department of Preventive Medicine and Public Health, School of Medicine, University of Navarra, C/Irunlarrea 1, 31008 Pamplona, Spain; 4 IdiSNA, Navarra Institute for Health Research, Pamplona, Spain; 5 Navarrabiomed-Miguel Servet Foundation, Pamplona, Spain; 6 Instituto de Salud Pública y Laboral de Navarra, Pamplona, Spain; 7 Centro de Investigación Biomédica en Red Área de Fisiología de la Obesidad y la Nutrición (CIBEROBN), Madrid, Spain; 8 Health Services Research on Chronic Patients Network (REDISSEC), Instituto de Salud Carlos III, Madrid, Spain

**Keywords:** Cardiovascular disease, Metabolic syndrome, Myocardial infarction, Stroke, Cardiovascular mortality, Mediterranean cohort, Cohort study

## Abstract

**Background:**

We aimed to investigate the association of metabolic syndrome (MetS) and its single components with cardiovascular risk and estimated their impact on the prematurity of occurrence of cardiovascular events using rate advancement periods (RAPs).

**Methods:**

We performed prospective analyses among 3976 participants (age range: 35–84, 55% female) in the Vascular Risk in Navarre (RIVANA) Study, a Mediterranean population-based cohort. MetS was defined based on the modified criteria of the American Heart Association/National Heart, Lung, and Blood Institute and the International Diabetes Federation. The primary endpoint was major cardiovascular event (a composite of myocardial infarction, stroke, or mortality from cardiovascular causes). Secondary endpoints were incidence of non-fatal myocardial infarction and non-fatal stroke, cardiovascular mortality, and all-cause mortality. Cox proportional hazards models, adjusted for potential confounders, were fitted to evaluate the association between MetS and its single components at baseline with primary and secondary endpoints.

**Results:**

During a median follow-up of 12.8 years (interquartile range, 12.5–13.1), we identified 228 primary endpoint events. MetS was associated with higher risk of incidence of major cardiovascular event, cardiovascular and all-cause mortality, but was neither associated with higher risk of myocardial infarction nor stroke. Compared with participants without MetS, the multivariable hazard ratio (95% confidence interval [CI]) among participants with MetS was 1.32 (1.01–1.74) with RAP (95% CI) of 3.23 years (0.03, 6.42) for major cardiovascular event, 1.64 (1.03–2.60) with RAP of 3.73 years (0.02, 7.45) for cardiovascular mortality, and 1.45 (1.17–1.80) with RAP of 3.24 years (1.21, 5.27) for all-cause mortality. The magnitude of the associations of the single components of MetS was similar than the predicted by MetS. Additionally, for each additional trait of MetS, incidence of major cardiovascular event relatively increased by 22% (1.22, 95% CI 1.09–1.36) with RAP of 2.31 years (0.88, 3.74).

**Conclusions:**

MetS was independently associated with CVD risk, cardiovascular and all-cause mortality. Components of the MetS were associated with similar magnitude of increased CVD, which suggests that MetS was not in excess of the level explained by the presence of its single components. Further research should explore the association of different combinations of the components of MetS with CVD.

## Background

Cardiovascular disease (CVD) is the most common cause of death globally and a significant contributor to morbidity, accounting for 17.8 million deaths worldwide, 330 million years of life lost, and 35.6 million years of disability [1, 2]. In addition to the burden of mortality and morbidity, CVD results in considerable economic costs, having an estimated impact of €210 billion year in the European Union economy [3].

Metabolic syndrome (MetS) refers to a constellation of physiological co-incident and inter-related risk factors that place an individual at high risk of developing CVD and diabetes mellitus type 2 [4, 5]. The main components of MetS are central obesity, elevated blood pressure, high triglycerides, low high density lipoprotein cholesterol (HDL cholesterol), and glucose intolerance. Despite the number of definitions proposed for MetS [5–8], the presence of at least three of the aforementioned risk factors is generally accepted to diagnose MetS.

Over the last decades, MetS has received particular attention due to its elevated prevalence in the general population. Approximately one-quarter of the world population has MetS [9], and the proportion of individuals with MetS is projected to continuously increase in parallel with the prevalence of obesity and diabetes type 2 [10, 11]. In Spain, the latest population-based study with representative national data estimated the prevalence of MetS in 22.7% [12].

A number of risk factors have been associated with MetS. Some authors have suggested that MetS lead to skeletal muscle abnormalities, and conversely, alteration in muscle pathology may precede and contribute to development of MetS [13]. Moreover, both low quality and quantity of skeletal muscles have been associated with the incidence and progression of MetS [14]. Other risk factors that have been associated with MetS were childhood retinol-binding protein 4 levels, independently of pediatric obesity [15], and fast eating speed, independently of total energy intake, body mass index at baseline, and body mass index change during the follow-up period [16]. On the other hand, an increase in relative skeletal muscle mass over time resulted to have a protective preventive effect on developing MetS [17]. Additionally, supervised high-intensity interval training has resulted to improve MetS and body composition in myocardial infarction patients with MetS undergoing cardiac rehabilitation [18].

Regarding the criteria used to define MetS, various studies have demonstrated an association between CVD with MetS independently of the definition of MetS [19–23]. These studies have generally reported a higher risk of developing CVD events for individuals with MetS. The largest systematic review and meta-analysis (n = 951,083) established in a twofold increase the risk of CVD, CVD mortality, and stroke, and a 1.5-fold increase the risk of all-cause mortality associated with MetS [24]. However, more recent studies have reported null or weak associations between MetS and CVD outcomes [25, 26]. Furthermore, the question whether MetS is a cardiovascular risk factor beyond the individual sum of all its single components remains controversial [27], and the risk of CVD has been reported to differ by the number of its traits [19]. In this context, epidemiological data regarding MetS and development of CVD in European countries is limited, particularly among Mediterranean populations. These populations are highly influenced by the Mediterranean dietary pattern — characterized by a high intake of monounsaturated and polyunsaturated fatty acids and other essential nutrients—which has been linked to a large number of health benefits, including CVD prevention and reduced mortality risk [28].

The aim of this study was to prospectively investigate data of a 13-year follow-up regarding the association of MetS and its single components with CVD risk and estimated the impact on prematurity of the occurrence of CVD using rate advancement periods (RAPs) in the RIVANA (Vascular Risk Study in Navarre) Study, a Mediterranean cohort of middle-aged adults. We additionally investigated to what extent the number of single components of MetS was associated with CVD.

## Methods

### Study population

The present study included data on participants in the RIVANA Study, a Mediterranean cohort with up to 13.6 years of follow-up. The cohort was designed to assess the prevalence of cardiovascular risk factors and MetS, their association with markers of subclinical atherosclerosis, and their impact on health outcomes in Navarre, one of the 17 Autonomous Communities of Spain. More specific details about the design, objectives, and methods of the RIVANA Study have been described elsewhere [29]. In brief, the RIVANA Study compromised a representative sample of 6553 participants (aged 35–84 years) that were randomly contacted to participate through the 2001 electoral register of Navarre. Eligibility criteria to enter the study included minimum age (35 years), residential area in Navarre, and non-institutionalization. Participants included in the electoral register that had died and subjects who were contacted multiple times with no response were excluded from the RIVANA Study. The recruitment of participants took place between June 2004 and December 2005. After application of inclusion and exclusion criteria, a total of 4,168 subjects agreed to participate in the study and were followed-up until December 2017. All the participants provided written informed consent to participate in the study and provided additional access to their medical records. The Institutional Review Board of the Government of Navarre approved the project protocol (approval code PI_2004/4).

For the present study, we excluded subjects with major CVD at baseline, namely prevalent myocardial infarction (n = 93) and stroke (n = 94), and individuals who were lost to follow-up (n = 5) (retention rate > 99%). The remaining 3,976 compromised the basis for our analyses.

### Definition of MetS

We used the most recent definition for the diagnosis of MetS, the harmonized definition of the American Heart Association/National Heart, Lung, and Blood Institute (AHA/NHLBI) and the International Diabetes Federation (IDF) with European origin specific waist circumference cut-off points [5]. Participants were classified with MetS if they had ≥ 3 of the following components: elevated waist circumference (abdominal circumference ≥ 94 cm for men and ≥ 80 cm for women); elevated blood pressure (systolic blood pressure ≥ 130 mmHg or diastolic blood pressure ≥ 85 mmHg) or antihypertensive medication use; elevated fasting glucose blood level (≥ 100 mg/dL) or antidiabetic agents or insulin use; reduced HDL-cholesterol (< 40 mg/dL for men and < 50 mg/dL for women) or receiving treatment for dyslipidemia; elevated triglycerides (≥ 150 mg/dL) or use of fenofibrate medication.

### Study outcomes

The primary endpoint of the study was a composite of myocardial infarction, stroke, and mortality from cardiovascular causes. Secondary endpoints were myocardial infarction, stroke, mortality from cardiovascular causes, and all-cause mortality. Mortality from cardiovascular causes included death from ischemic heart disease, heart failure, sudden cardiac death, death due to stroke, peripheral vascular disease, and death due to other cardiovascular causes (Additional file 1: Appendix S1). We identified endpoints of the study by record linkage with four different sources of information: (a) the Assisted Morbidity Registry of Navarre, which includes the Minimum Basic Data Set with information of all hospital discharges (both public and private) in the Navarre Health System; (b) the Navarre primary care electronic-health records database; (c) the Regional Registry of Myocardial infarction of Navarre; and (d) the National Statistics Institute of Spain to identify deceased cases and determine the cause of death. The endpoints of the study were examined by members of the study team, who classified then into primary or secondary based on the criteria of Additional file 1: Appendix S1. Additionally, all endpoint events of the study were confirmed by two investigators through access to patients’ electronic-health records. The primary care electronic-health records database in the community of Navarre covers around > 97% of the population (approximately 650,000 inhabitants). This database was designed to account for diverse episodes and health conditions, and was coded according to the International Classification of Primary Care, Second Edition (ICPC-2) [30]. The database has been previously validated with other disease diagnoses such as diabetes type 2 and childhood obesity, resulting in a valid source for epidemiological surveillance [31, 32]. Moreover, in Spain, with a universal healthcare system, primary health care is frequently used by the Spanish population (in 2016, the frequency of medical consultations was 5.2 visits per person/year [33]). The use of electronic-health records database and the high frequency of primary care consultations allowed us to have a strict follow-up of the participants of the study (< 1% losses to follow-up). Moreover, the use of the four aforementioned combined sources of information to identify endpoints of the study, which were supported by the Navarre and/or the Spanish Government, can be assumed to have a 100% positive predictive value for cardiovascular events included in the analysis.

### Other covariates

Trained nurses, who had previously received interview-technique training, collected participants’ information on sociodemographic factors, lifestyle (including smoking, alcohol consumption, physical activity, and adherence to Mediterranean diet), family and personal medical history via face-to-face structured interviews. A structured questionnaire was used to collect patients’ information. The questionnaire was designed by a group of experts and was previously piloted in a subsample of participants. The participants’ information gathered in the questionnaire was reviewed by two investigators and encoded to protect patients’ confidentiality. Anthropometric measures (height, weight, and waist circumference) were measured in triplicate by registered nurses using standardized techniques, calibrated scales, and wall-mounted stadiometers, respectively. Waist circumference was measured in duplicate by trained staff, halfway between the last rib and the iliac crest using an anthropometric tape parallel with the floor [34]. Blood pressure was measured in triplicate using a validated semiautomatic oscillometer (OMRON® M4-1). Measurement averages were calculated later for analysis purposes. Blood sample were collected to determine biological parameters (fasting glucose, HDL- cholesterol, plasma triglycerides, uric acid, plasma C-reactive protein, plasma creatinine, among others). Urine samples were additionally collected to determine creatinine and microalbumina. Prevalent CVD was self-reported and included any history of angina, coronary artery bypass surgery or other revascularization procedure, heart failure, or peripheral disease. Adherence to Mediterranean diet was assessed with a validated 14-point Mediterranean diet questionnaire [35, 36]. Duration and intensity of physical activity was self-reported using the validated Spanish version of the *Minnesota Leisure Time Physical Activity Questionnaire* [37, 38]. To calculate metabolic equivalent hours per week (METs-h/week), we multiplied the time (in hours) spent in each activity per week by its intensity (METs-h) based on the compendium of physical activity [39].

### Statistical analysis

The baseline characteristics of participants were described according to the presence or absence of MetS. Continuous variables were expressed as mean ± standard deviation (SD), and categorical data were summarized as the number of participants and percentages. Chi-square tests and Student’s t-test were used to assess baseline differences between non-MetS and MetS participants.

Cox proportional hazards models were conducted to examine the association of MetS with the cardiovascular outcomes of the study. We considered time in the study as the interval between the date of completion of the baseline interview and last recorded follow-up, the date of diagnosis of the first stroke, first myocardial infarction, or the date of death, whichever occurred first. Participants without cardiovascular events exceeding the end of follow-up date (31 December 2017) were truncated at this time. All the models included age as underlying time variable, and we stratified the models by age at recruitment (deciles). We fitted multivariable-adjusted Cox regression models for the following baseline covariates: alcohol (never, sometimes, regularly), educational level (primary or less, secondary, college/university), low density lipoprotein cholesterol (LDL cholesterol, continuous), Mediterranean diet adherence score (continuous), physical activity (METs-h/week, continuous), prevalent cardiovascular disease (dichotomous), renal disease (dichotomous), sex (dichotomous), and smoking status (never, current, and former smoker). To determine whether the MetS had an additional value in the risk of CVD beyond its single components, we additionally calculated hazard ratios (HRs) and its 95% confidence intervals (CI) for the association between the individual components of MetS and the endpoints of the study.

The effect of MetS with respect to the time dimension of premature cardiovascular events of the study was assessed with estimates of RAPs [40, 41]. In other words, RAP measures the effect of an exposure in terms of time period by which exposed population would experience the same disease risk as the non-exposed population, in the absence of competing risks. RAPs have been shown to be an informative measure to report the impact of a risk factor on chronic disease occurrence. We calculated point estimates of RAP from the ratio of the adjusted HRs for the exposure of our study and the HR for age obtained in the multivariable Cox models, and 95% CI were derived. For RAPs calculations, Cox regression models included age as continuous variable (instead of underlying time variable) and were adjusted for the same potential confounders previously described. We verified the proportionality of hazard assumption using Schoenfeld residuals [42] to correctly interpret RAP estimates [43].

Multivariable-adjusted models were additionally conducted to explore the association between the number of traits (0–1 trait, 2 traits, 3 traits, 4 traits, and 5 traits) and the different endpoints of the study, and were represented in Kaplan–Meier cumulative-incidence curves. We performed this categorization of the number of traits because of the very low number of subjects with 0 traits who experienced endpoint events.

The interaction between MetS and age (continuous and below or above median [53 years]), sex (dichotomous), and Mediterranean diet adherence (continuous) in the association of MetS and the endpoint events of the study were assessed by testing an interaction product-term with the likelihood ratio test.

Sensitivity analyses were performed by re-running our analyses using the harmonized definition of the IDF and AHA/NHLBI with specific cut-off points for the criteria of waist circumference proposed for Spanish population [44], and using the National Cholesterol Education Program (NCEP) Adult Treatment Panel-III (ATP-III) definition [6]. Specific waist circumference cut-off points for Spanish population were proposed by Martínez-Larrad et al. [44] and set at 94.5 cm for men and 89.5 cm for women. The harmonized criteria (using specific cut-off points for European population) and the NCEP-ATP III definition principally differ in their threshold for impaired fasting glucose (100 mg/dL *vs.* 110 mg/dL, respectively) and for waist circumference (≥ 94 cm *vs.* ≥ 102 cm for men and ≥ 80 cm vs*.* ≥ 88 cm for women). All analyses were performed with STATA version 14 (StataCorp LP), and a 2-sided p-value smaller than 0.05 was deemed as statistical significance. Missing data were imputed with regression equations to predict missing covariates. Imputations represented < 1% of missing covariates (Additional file 1: Appendix S2), and there was no missing information of any of the components of MetS.

## Results

**Fig. 1:**
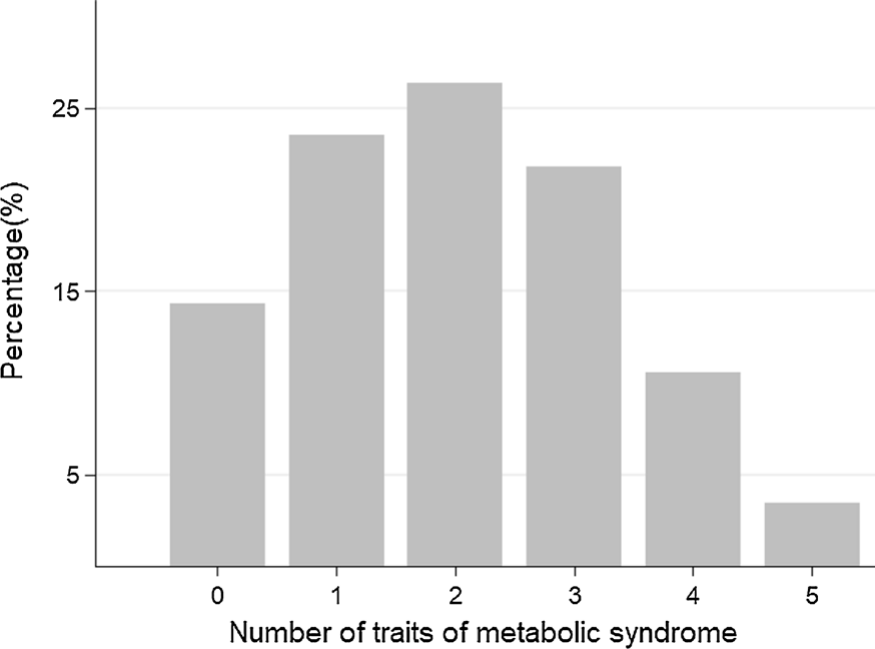
Distribution of participants of the study by number of traits of metabolic syndrome according to the IDF and AHA/NHLBI definition

The baseline characteristic of participants according to the harmonized definition of MetS of the IDF and AHA/NHLBI [5] are summarized in Table 1. Overall, 1424 (35.8%) participants had MetS (45.7% of overall men and 27.9% of overall women). In comparison with participants without MetS, participants with MetS were older, mostly male, had a lower educational level, were more likely to smoke, less physically active, and showed a poorer adherence to the Mediterranean diet. As expected, participants with MetS had a higher prevalence of diabetes, hypertension, hypercholesterolemia, and generally worse control of risk factors at baseline. Among all participants, elevated waist circumference (69%) was the most predominant trait, followed by elevated blood pressure (56%), elevated fasting glucose (40%), elevated triglycerides (19%), and reduced HDL-cholesterol (17%). When examining the overall population of the study per the number of traits, we found that the most common combination was having 2 traits (26.3%), followed by having 1 trait (23.5%), 3 traits (21.8%), 0 traits (14.3%), 4 traits (10.6%), and having 5 traits (3.5%) (Fig. 1).

**Table 1:** Baseline characteristics of study participants of the Rivana (Vascular Risk in Navarre) according the definition of metabolic syndrome of the international Diabetes Federation and the AHA/National Heart, Lung, and Blood Institute (n = 3976)

Characteristics	Overall participants	No metabolic syndrome	Metabolic syndrome	p-value
N (frequency)	3976	2552 (64.2%)	1424 (35.8%)	
Age, years	53.3 ± 12.4	50.2 ± 11.6	58.8 ± 11.9	< 0.001
Sex, men	1775 (45%)	964 (38%)	811 (57%)	< 0.001
Higher level of attained education				< 0.001
Primary or less	2177 (55%)	1211 (47%)	966 (68%)	
Secondary	906 (23%)	663 (26%)	243 (17%)	
College/university	893 (23%)	678 (27%)	215 (15%)	
BMI, kg/m^2^	26.9 ± 4.5	25.4 ± 3.8	29.7 ± 4.3	< 0.001
Physical activity, METs-h/week	44 ± 38	45 ± 39	42 ± 37	0.031
Smoking status				0.026
Never	1139 (29%)	377 (26%)	377 (26%)	
Current	1559 (39%)	965 (38%)	594 (42%)	
Former smokers	1278 (32%)	825 (32%)	453 (32%)	
Alcohol consumption				< 0.001
Never	1174 (28%)	713 (27%)	461 (30%)	
Sometimes	1055 (25%)	737 (28%)	318 (21%)	
Regularly	1933 (46%)	1163 (45%)	770 (50%)	
MedDiet	8.7 ± 2.0	8.6 ± 2.0	8.8 ± 2.0	0.003
Medications				
Antihypertensive therapy	676 (17%)	175 (7%)	501 (35%)	< 0.001
Lipid-Lowering therapy	380 (10%)	52 (2%)	328 (23%)	< 0.001
Antidiabetic agents	156 (4%)	21 (1%)	135 (9%)	< 0.001
Risk factors				
Weight, kg	72 ± 14	68 ± 12.2	79 ± 14	< 0.001
Waist circumference, cm	92 ± 13	88 ± 12	101 ± 11	< 0.001
Systolic BP, mmHg	132 ± 19	126 ± 17	144 ± 17	< 0.001
Diastolic BP, mmHg	80 ± 10.2	77 ± 9	85 ± 10	< 0.001
Fasting blood glucose, mg/dL	101 ± 22	94 ± 12	114 ± 28	< 0.001
Total cholesterol, mg/dL	213 ± 38	210 ± 37	217 ± 39	< 0.001
LDL cholesterol, mg/dL	127 ± 34	125 ± 33	131 ± 35	< 0.001
HDL cholesterol, mg/dL	64 ± 17	68 ± 16	57 ± 15	< 0.001
Ratio TC: HDL-c (× 100)	3.5 ± 1.1	3.2 ± 0.9	4.0 ± 1.2	< 0.001
Triglycerides, mg/dL	113.0 ± 82	89 ± 45	156 ± 111	< 0.001
TyG index^a^	8.5 ± 0.6	8.2 ± 0.4	8.9 ± 0.6	< 0.001
TyG-WC^b^	787 ± 1423	722 ± 112	903 ± 117	< 0.001
Self-reported chronic and cardiovascular disease				
Diabetes	355 (9%)	87 (3%)	268 (19%)	< 0.001
Hypertension	997 (25%)	349 (14%)	648 (46%)	< 0.001
Hypercholesterolemia	1494 (38%)	760 (30%)	734 (52%)	< 0.001
Angina pectoris	57 (1%)	13 (1%)	44 (3%)	< 0.001
Heart failure	45 (1%)	13 (1%)	32 (2%)	< 0.001
Surgery or other revascularization procedure	30 (1%)	9 (0.4%)	21 (1%)	< 0.001
Peripheral vascular disease	57 (1%)	25 (1%)	32 (2%)	0.001
C-Reactive protein, mg/dL	9.2 ± 27.6	7.5 ± 27.8	12.0 ± 27.0	< 0.001
Microalbuminuria^c^	231 (6%)	93 (4%)	138 (10%)	< 0.001
Proteinuria^d^	42 (1%)	17 (1%)	25 (2%)	0.001
Renal disease^e^	46 (1%)	22 (1%)	24 (2%)	0.002
Metabolic syndrome traits				
Elevated waist circumference^f^	2755 (69%)	1389 (54%)	1366 (96%)	< 0.001
Elevated blood pressure^g^	2235 (56%)	935 (37%)	1300 (91%)	< 0.001
Elevated fasting glucose^h^	1595 (40%)	456 (18%)	1139 (80%)	< 0.001
Reduced HDL-cholesterol^i^	665 (17%)	109 (4%)	556 (39%)	< 0.001
Elevated triglycerides^j^	745 (19%)	140 (5%)	605 (42%)	< 0.001

Values are means ± SD or numbers of participants (percentages)

*BMI *body mass index, *BP* blood pressure, *Med Diet* 14-item Mediterranean diet score, *LDL* low density lipoprotein, *HDL* high density lipoprotein, *MET* metabolic equivalent, *Ratio TC*: HDL-c ratio total cholesterol, *HDL* cholesterol; *TyG index* triglycerides and glucose index, *TyG-WC* product of triglycerides and glucose and waist circumference

^a^TyG index: Ln(triglycerides × fasting glucose/2)

^b^TyG-WC: product of the TyG index and waist circumference

^c^Microalbuminuria: ≥ 30 to < 300 mg/dL urine albumin

^d^Proteinuria: ≥ 300 mg/dL urine albumin

^e^Renal disease: serum creatinine ≥ 1.5 mg/dL for men or ≥ 1.4 mg/dl for women

^f^Elevated waist circumference (abdominal perimeter ≥ 80 cm for men and ≥ 94 cm for women)

^g^Elevated blood pressure (systolic blood pressure ≥ 130 mmHg or diastolic blood pressure ≥ 85 mmHg or receiving antihypertensive medication treatment)

^h^Elevated fasting glucose (≥ 100 mg/dL) or receiving antidiabetic treatment with insulin or oral hypoglycemic agents

^i^Reduced HDL-cholesterol (< 40 mg/dL for men and < 50 mg/dL for women) or receiving treatment for dyslipidemia

^j^Elevated triglycerides, triglycerides ≥ 150 mg/dL or receiving fenofibrate treatment

A total of 3,976 men and women initially free of major CVD were followed-up for a median time of 12.8 years (interquartile range 12.5 to 13.1). Among the total number of participants, 228 subjects experienced the primary composite endpoint, which represented an incidence rate of 4.77 per 1000 person-years (2.98 and 8.15 for the non-MetS and MetS group, respectively). For secondary endpoints, we accounted for 80 myocardial infarction events, 96 stroke events, 85 deaths from CVD causes, and 381 all-cause deaths. Detailed information about causes of deaths is provided in Additional file 1: Appendix S3.

Estimates of HR and RAP for the endpoint events of the study for the association with metabolic syndrome and its components are shown in Table 2. RAP represents the baseline age difference at which exposed subjects (participants with MetS) reach the same risk of disease (endpoint events) as unexposed subjects (non-MetS participants). After multiple-adjustment, MetS was associated with higher risk of major cardiovascular event with HRs of 1.32 (1.01–1.74) and a RAP (95% CI) of 3.23 years (0.03, 6.42). Furthermore, elevated blood pressure, reduced HDL-cholesterol, and elevated triglycerides were associated with higher risk of major cardiovascular event. For secondary endpoint events, MetS was significantly associated with higher mortality from cardiovascular disease 1.64 (95% CI, 1.03–2.60) with RAP of 3.73 years (95% CI 0.02, 7.45), and higher all-cause mortality 1.45 (95% CI, 1.17–1.80) with RAP of 3.24 years (95% CI 1.21, 5.27) but was not associated with higher risk of myocardial infarction, or stroke. Complete results of the association for primary and secondary endpoints are shown in Table 2.

**Table 2:** Estimates of cardiovascular events for the association with metabolic syndrome and its components in the Rivana cohort (n = 3,976)

	Primary endpoint		Secondary endpoints							
	Myocardial infarction, stroke, and mortality from cardiovascular disease		Myocardial infarction		Stroke		Mortality from cardiovascular disease		All-cause mortality	
Cases	228		80		96		85		381	
Person- years of follow up	47,838		48,215		48,227		48,629		48,629	
Incidence rate/1000 person-year	4.77		1.66		1.99		1.75		7.83	
	HR (95% CI)^a^	RAP (95% CI)	HR (95% CI)^a^	RAP (95% CI)	HR (95% CI)^a^	RAP (95% CI)	HR (95% CI)^a^	RAP (95% CI)	HR (95% CI)^a^	RAP (95% CI)
Metabolic syndrome and its traits^b^										
Metabolic syndrome	*1.32 (1.01–1.74)*	*3.23 (0.03, 6.42)*	1.15 (0.73–1.82)	2.46 (-4.99, 9.90)	1.16 (0.76–1.76)	1.68 (− 2.64, 6.01)	*1.64 (1.03–2.60)*	*3.73 (0.02, 7.45)*	*1.45 (1.17–1.80)*	*3.24 (1.21, 5.27)*
Elevated waist circumference	1.24 (0.87–1.77)	2.28 (− 1.76, 6.32)	1.54 (0.83–2.85)	7.29 (-3.18, 17.76)	1.26 (0.72–2.21)	2.60 (− 3.16, 8.37)	0.90 (0.50–1.61)	− 1.00 (− 5.45, 3.44)	1.37 (1.02–1.83)	2.15 (− 0.49, 4.79)
Elevated blood pressure	*1.63 (1.12–2.35)*	*6.07 (1.35, 10.80)*	0.95 (0.56–1.61)	-0.35 (-8.52, 7.83)	*2.16 (1.15–4.06)*	*8.98 (1.29, 16.67)*	*2.59 (1.16–5.75)*	*7.60 (0.68, 14.52)*	*1.45 (1.08–1.93)*	*3.21 (0.46, 5.96)*
Elevated fasting glucose	1.20 (0.91–1.57)	2.06 (− 1.06, 5.18)	0.89 (0.56–1.42)	-1.13 (-8.20, 5.94)	1.55 (1.00–2.40)	*4.85 (0.13, 9.58)*	1.35 (0.86–2.13)	2.08 (− 1.47, 5.63)	1.22 (0.99–1.51)	1.83 (− 0.12, 3.78)
Reduced HDL-cholesterol	*1.42 (1.06–1.92)*	*3.95 (0.54, 7.37)*	*1.68 (1.01–2.78)*	7.90 (-0.57, 16.37)	1.10 (0.68–1.79)	0.81 (− 4.00, 5.63)	*1.67 (1.04–2.66)*	*4.03 (0.38, 7.68)*	*1.35 (1.07–1.70)*	*2.35 (0.26, 4.44)*
Elevated triglycerides	*1.38 (1.02–1.85)*	*3.41 (0.06, 6.75)*	*1.76 (1.09–2.84)*	*8.48 (0.58, 16.38)*	0.96 (0.58–1.59)	− 0.34 (− 5.34, 4.65)	1.34 (0.82–2.20)	2.20 (− 1.63, 6.03)	*1.42 (1.13–1.79)*	*3.30 (1.22, 5.39)*

*CI* confidence interval, *HDL* high density lipoprotein, *HR* hazard ratio, *RAP* rate advancement period

Italic values are statistically significant at p-value < .05

^a^Model adjusted for age (underlying variable), alcohol (never, sometimes, regularly), higher level of attained education (primary or less, secondary, college/university), LDL cholesterol level (continuous), Mediterranean diet adherence (continuous), physical activity (metabolic equivalent minutes per day, continuous), prevalent cardiovascular disease (dichotomous), renal disease (dichotomous), sex, smoking status (never, current, and former smoker), and stratified by age (in deciles)

^b^Number of participants with metabolic syndrome (n = 1,424); with elevated waist circumference (n = 2,755); with elevated blood pressure (n = 2,235); with elevated fasting glucose (n = 1,595); with reduced HDL-cholesterol (n = 665); with elevated triglycerides (n = 745)

**Fig. 2:**
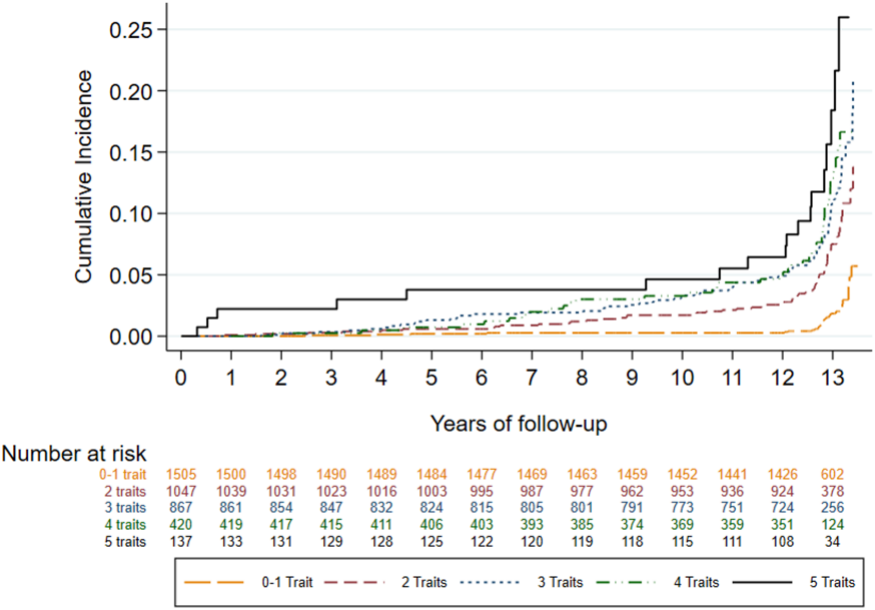
Kaplan–Meier estimates of the cumulative incidence of primary endpoint events—myocardial infarction, stroke, or mortality from cardiovascular causes—according to the number of metabolic syndrome traits (0–1 trait, 2 traits, 3 traits, 4 traits, and 5 traits) in the Rivana cohort (n = 3976)

The association between number of traits and the risk of cardiovascular endpoint events and estimates of RAPs are presented in Table 3. Overall, the risk of incidence of cardiovascular events significantly increased for each additional trait of MetS, except for myocardial infarction. These findings were confirmed when representing Kaplan–Meier cumulative incidence curves (Fig. 2  and Additional file 1: Appendix S4).

**Table 3:** Estimates of cardiovascular events for the association with metabolic syndrome according to the number of traits in the Rivana cohort (n = 3,976)

	Primary endpoint		Secondary endpoints							
	Myocardial infarction, stroke, and mortality from cardiovascular disease		Myocardial infarction		Stroke		Mortality from cardiovascular disease		All-cause mortality	
Cases	228		80		96		85		381	
Person- years of follow up	47,838		48,215		48,227		48,629		48,629	
Incidence rate/1000 person-year	4.77		1.66		1.99		1.75		7.83	
	HR (95% CI)^a^	RAP (95% CI)	HR (95% CI)^a^	RAP (95% CI)	HR (95% CI)^a^	RAP (95% CI)	HR (95% CI)^a^	RAP (95% CI)	HR (95% CI)^a^	RAP (95% CI)
Number of traits^b^										
0–1 trait	1.00 (ref.)	0.00 (ref.)	1.00 (ref.)	0.00 (ref.)	1.00 (ref.)	0.00 (ref.)	1.00 (ref.)	0.00 (ref.)	1.00 (ref.)	0.00 (ref.)
2 traits	*1.87 (1.17–2.98)*	*7.44 (1.67, 13.22)*	1.24 (0.63–2.43)	3.84 (− 7.24, 14.92)	*3.02 (1.37–6.66)*	*12.08 (2.79, 21.38)*	2.36 (0.88–6.35)	6.64 (− 1.45, 14.73)	*1.56 (1.07–2.25)*	3.46 (− 0.03, 6.96)
3 traits	*1.85 (1.16–2.95)*	*7.31 (1.49, 13.14)*	1.01 (0.50–2.05)	0.65 (− 10.73, 12.02)	*2.46 (1.10–5.50)*	*9.99 (0.65, 19.34)*	*3.06 (1.16–8.02)*	8.62 (0.60, 16.65)	*1.87 (1.30–2.68)*	*5.17 (1.71, 8.63)*
4 traits	*2.06 (1.24–3.42)*	*8.38 (2.00, 14.76)*	1.62 (0.77–3.43)	7.78 (− 5.13, 20.68)	2.36 (0.98–5.68)	9.77 (− 0.31, 19.84)	*3.06 (1.11–8.45)*	8.54 (0.09, 16.99)	*1.88 (1.27–2.79)*	*5.40 (1.63, 9.17)*
5 traits	*3.08 (1.67–5.69)*	*13.51 (5.80, 21.23)*	2.36 (0.94–5.92)	15.18 (− 1.05, 31.41)	*4.13 (1.51–11.24)*	*15.41 (3.77, 27.06)*	*4.20 (1.25–14.04)*	*10.99 (1.11, 20.87)*	*2.82 (1.74–4.57)*	*8.80 (4.17, 13.42)*
Per additional trait	*1.22 (1.09–1.36)*	*2.31 (0.88, 3.74)*	1.18 (0.98–1.42)	2.88 (− 0.58, 6.35)	*1.20 (1.01–1.43)*	*2.00 (0.04, 3.96)*	*1.28 (1.06–1.56)*	*1.88 (0.28, 3.49)*	*1.22 (1.11–1.33)*	*1.71 (0.84, 2.58)*

*CI* confidence interval, *HR* hazard ratio, *RAP* rate advancement period, *ref* reference

Italic values are statistically significant at p-value < .05

^a^ Model adjusted for age (underlying variable), alcohol (never, sometimes, regularly), higher level of attained education (primary or less, secondary, college/university), LDL cholesterol level (continuous), Mediterranean diet adherence (continuous), physical activity (metabolic equivalent minutes per day, continuous),prevalent cardiovascular disease (dichotomous), renal disease (dichotomous), sex, smoking status (never, current, and former smoker), and stratified by age (in deciles)

^b^Number of participants with 0–1 trait (n = 1,505); with 2 traits (n = 1,047); with 3 traits (n = 867); with 4 traits (n = 420); with 5 traits (n = 137)

We did not find any interaction between MetS and age (continuous, *P*-interaction = 0.089; categorical [53 years], *P*-interaction = 0.313), sex (*P-*interaction = 0.602), or Mediterranean diet score (continuous, *P*-interaction = 0.853) in the association with the primary endpoint of the study. No interactions between MetS and sex, age, or Mediterranean diet adherence for the rest of endpoint events of the study were statistically significant.

To assess the robustness of our results, we re-run our analyses using different criteria: the IDF and AHA/NHLBI definition with specific waist circumference cut-off points for Spanish population, and the NCEP-ATP III definition of MetS. Overall, estimates of HR and RAP for the incidence of the primary point events with their association with MetS and its components remained similar. Nevertheless, the magnitude of the association per number of traits with MetS according to the NCEP-ATP III definition was slightly different (Table 4).

**Table 4:** Sensitivity analyses. Estimates of primary endpoint events—myocardial infarction, stroke, or mortality from cardiovascular causes—for the association with metabolic syndrome and its components in the Rivana cohort (n = 3976) according to the NCEP-ATPIII definition [6] and specific waist circumference cut-off points previously developed for the Spanish population [32]

	Primary endpoint (myocardial infarction, stroke, and mortality from cardiovascular disease)				
	IDF and AHA/NHLBI definition with specific waist circumference cut-off points for Spanish population^a^		NCEP-ATPIII Metabolic Syndrome definition^b^		
Cases	228		228		
Person- years of follow up	47,838		47,838		
Incidence rate/1000 person-year	4.77		4.77		
	HR (95% CI)^c^	RAP (95% CI)	HR (95% CI)^c^		RAP (95% CI)
Metabolic syndrome and components^d, e^					
Metabolic syndrome	*1.39 (1.05–1.83)*	*3.64 (0.27, 7.01)*	*1.36 (1.03–1.78)*		*3.33 (0.10–6.56)*
Elevated waist circumference	*1.40 (1.03–1.90)*	3.66 (0.03, 7.29)	1.30 (0.98–1.72)		2.79 (− 0.44, 6.01)
Elevated blood pressure	*1.64 (1.12–2.39)*	*6.20 (1.37, 11.02)*	*1.63 (1.12–2.35)*		*6.07 (1.35, 10.80)*
Elevated fasting glucose	1.21 (0.92–1.58)	2.17 (− 0.93, 5.26)	1.25 (0.94–1.66)		2.23 (− 1.03, 5.50)
Reduced HDL-cholesterol	*1.39 (1.02–1.89)*	*3.68 (0.06, 7.29)*	*1.42 (1.06–1.92)*		*3.95 (0.54, 7.37)*
Elevated triglycerides	*1.38 (1.02–1.85)*	3.43 (− 0.03, 6.88)	*1.38 (1.02–1.85)*		*3.41 (0.06, 6.75)*
Number of traits^d, e^					
0–1 trait	1.00 (ref.)	0.00 (ref.)	1.00 (ref.)		0.00 (ref.)
2 traits	*1.62 (1.06–2.47)*	*5.91 (0.65, 11.17)*	1.39 (0.98–1.96)		3.51 (− 0.57, 7.59)
3 traits	*1.66 (1.08–2.57)*	*6.02 (0.54, 11.50)*	1.34 (0.92–1.97)		3.11 (− 1.42, 7.64)
4 traits	*2.06 (1.30–3.26)*	*8.42 (2.49, 14.35)*	*2.03 (1.31–3.15)*		*7.74 (2.39, 13.09)*
5 traits	*2.57 (1.38–4.81)*	*11.48 (3.64, 19.32)*	*2.33 (1.19–4.58)*		*9.78 (1.82, 17.80)*
Per additional trait	*1.22 (1.10–1.36)*	*2.38 (0.96, 3.80)*	*1.22 (1.09–1.36)*		*2.31 (0.88, 3.74)*

*CI* confidence interval, *HDL* high density lipoprotein, *HR* hazard ratio, *RAP* rate advancement period

^a^Metabolic syndrome according to the harmonized definition of the International Diabetes Federation (IDF) American Heart Association/National Heart, Lung, and Blood Institute (AHA/NHLBI) with specific waist circumference cut-off points for Spanish population: 94.5 cm for men and 89.5 cm for women [32]

^b^Metabolic syndrome according to the National Cholesterol Education Program (NCEP) Adult Treatment Panel-III (ATP-III) definition [6]

Italic values are statistically significant at *p-*value < .05

^c^Model adjusted for age (underlying variable), alcohol (never, sometimes, regularly), higher level of attained education (primary or less, secondary, college/university), LDL cholesterol level (continuous), Mediterranean diet adherence (continuous), physical activity (metabolic equivalent minutes per day, continuous), prevalent cardiovascular disease (dichotomous), renal disease (dichotomous), sex, smoking status (never, current, and former smoker), and stratified by age (in deciles)

^d^Number of participants according to the harmonized definition of the *IDF and AHA/NHLBI* with specific waist circumference cut-off points for Spanish population with metabolic syndrome (n = 1,147); with elevated waist circumference (n = 1,630); with elevated blood pressure (n = 2,235); with elevated fasting glucose (n = 1,595); with reduced HDL-cholesterol (n = 665); with elevated triglycerides (n = 745). Number of participants with 0–1 trait (n = 1,879); with 2 traits (n = 950); with 3 traits (n = 710); with 4 traits (n = 327); with 5 traits (n = 110)

^e^Number of participants according to the *NCEP-ATPIII* definition with metabolic syndrome (n = 839); with elevated waist circumference (n = 1,490); with elevated blood pressure (n = 2,235); with elevated fasting glucose (n = 740); with reduced HDL-cholesterol (n = 665); with elevated triglycerides (n = 745). Number of participants with 0–1 trait (n = 2,205); with 2 traits (n = 932); with 3 traits (n = 538); with 4 traits (n = 226); with 5 traits (n = 75)

## Discussion

We aimed to prospectively investigate the prevalence and risk estimates of MetS and its components with major cardiovascular events in a Mediterranean cohort of around 4,000 middle-aged adult participants with 12.8 years of median of follow-up. After multivariable-adjustment, we found that MetS was associated with higher incidence of primary endpoint of the study—a composite of myocardial infarction, stroke, and mortality from cardiovascular causes. Furthermore, elevated blood pressure, reduced HDL-cholesterol, and elevated triglycerides were associated with a similar magnitude of increased CVD, which suggests that MetS was not in excess of the level explained by the presence of its single components. Regarding the magnitude of the RAP, we found that participants with MetS will reach the same CVD risk level as individuals without MetS about 3.2 years earlier. Additionally, the risk of CVD monotonically increased across higher number of cardiovascular risk factors.

### Metabolic syndrome (MetS)

We found a significant association between MetS and major CVD incidence. The magnitude of the association was smaller than the twofold increase in risk reported in the largest meta-analyses of cardiovascular risk and metabolic syndrome [24]. In this meta-analyses, the authors additionally reported significant associations for myocardial infarction, stroke, CVD mortality, and all-cause mortality. In turn, we did not find association between MetS with myocardial infarction or stroke. Our findings are in line with previous literature in which the association between MetS and CVD outcomes resulted relatively weak or null [25, 26, 45–47].

We additionally found that individual traits of the syndrome were independently associated with diverse cardiovascular outcomes, which is consistent with prior research [25, 48, 49]. The magnitude of the association of the single components of MetS was similar than the predicted by the actual MetS. Moreover, individual traits were independently associated with myocardial infarction or stroke, whereas the association with MetS resulted non-significant, questioning the predict value of MetS beyond its single components [25]. However, some authors have argued that the view of the MetS as a predictive tool is too simplistic [50, 51]. These authors pointed out that MetS is a biological construct of coexistent components that share common physiopathological pathways, and their mechanisms of actions may partially overlap, resulting in a total combined effect lower than the summed of the single components.

The findings of this study were consistent with previous findings of the RIVANA cohort, in which individual traits of the syndrome were more determinant than the actual construct of MetS in the association of asymptomatic CVD and epicardial adipose tissue [52, 53]. Moreover, we found that for each additional trait of MetS, incidence of cardiovascular events significantly increased, except for myocardial infarction events. Our findings call into question the utility of categorizing individuals as having or not having MetS, which is an ultimate classification by number of single components (having < 3 or ≥ 3 MetS traits). This categorization may produce misleading messages if the concept of MetS is only used to identify patients at increased risk, as we demonstrated individuals without MetS may also stay at high risk of developing cardiovascular events. In our study the risk of myocardial infarction did not significant increased per additional trait, which suggests that the risk associated with myocardial infarction events may vary depending on the different combinations of its single components.

### Rate advancement periods (RAPs)

We additionally calculated estimates of RAPs of endpoint events to illustrate the impact of MetS and its components. As described previously, this measure expresses the impact of a risk factor on the timing of disease occurrence and is similar to the concept of years of potential life lost [41]. For the general population, epidemiological measures may be challenging to understand. RAP results in a more understandable message and may increase motivation for behavior change. Previous studies have used RAPs to report the impact of dietary patterns [54], lifestyle-related factors [55], and the benefits of quitting smoking on health [56]. Our results advocated for a proportion of excess of major cardiovascular risk of 3.2 years attributable to MetS and up to 6.1 years attributable to elevated blood pressure. In other words, and based on our results, an individual with 60-year-old and MetS has the same risk of major cardiovascular events than a 63-year-old participant without MetS. In the case of a 60-year-old with elevated blood pressure, the risk of suffering major cardiovascular events is comparable to the risk of a subject who is 66 years old with normal levels of blood pressure. The greatest excess of cardiovascular risk resulted to be 9.0 years for stroke, attributable to elevated blood pressure.

### Limitations and strengths

We acknowledge that our study has some limitations. First, despite thorough adjustments for sociodemographic, lifestyle, and dietary risk factors such as smoking, alcohol, educational level, Mediterranean diet adherence, and physical activity, potential residual confounding cannot be completely ruled out. Second, covariate information was collected at baseline, and participants might change their habits and lifestyle during the follow-up. Third, the number of some endpoint events of our cohort was limited, leading to wide confidence intervals and, consequently, may have attenuated our statistical power; yet, previous findings from the RIVANA cohort related to MetS have showed consistently reliable results [52, 53]. Fourth, the RIVANA cohort has been designed to be representative of the Navarre population, and our result may only allow us to generalize the results to Mediterranean middle-aged adults; nevertheless, it seems to be plausible that the results might be generalized to other European and Caucasian populations.

Despite the aforementioned limitations, the strengths of this study rely on the population-based nature of the cohort that includes a representative population between 35 and 84 years, the long follow-up (median 12.8 years, interquartile range 12.5 to 13.1), very high retention rate (> 99%), the rigorous method of collection of cardiovascular events supported by public resources, the use of an electronic-health records database that has been previously validated with other health conditions [31, 32], and the inclusion of sensitivity analyses with specific waist circumference cutoff levels for Spanish population [44].

## Conclusions

MetS was found to be independently associated after adjusting for multiple potential confounders with the incidence of CVD, mortality from CVD, and all-cause mortality, but not with myocardial infarction or stroke. Single components of the MetS were independently associated with all the endpoints of the study, with similar magnitudes than MetS itself. We observed that the risk of cardiovascular events increased with higher number of MetS components, except for myocardial infarction, which suggests that the risk may vary depending on the combinations of its single components. Further research should examine the association of different combinations of traits of MetS and their association with cardiovascular endpoints.

## Supplementary information

**Additional file 1: Appendix S1.** Diagnostic criteria for endpoints of the study. **Appendix S2.** Percentages of imputed information for imputed variables. **Appendix S3.** Causes of death, including coronary heart disease, cerebrovascular disease, other cardiovascular causes, other non-cardiovascular causes, cancer, and unknown causes, among the participants of the Rivana (Vascular Risk in Navarre) cohort (n = 3,976). **Appendix S4.** Kaplan-Meier estimates of the cumulative incidence of A) myocardial infarction, B) stroke, C) mortality from cardiovascular causes, and D) all-cause mortality according to the number of metabolic syndrome traits (0-1 trait, 2 traits, 3 traits, 4 traits, and 5 traits) in the Rivana cohort (n = 3,976).  

## Data Availability

The datasets during and/or analyzed during the current study are available from the corresponding author on reasonable request.

## Abbreviations

AHA/NHLBI: American Heart Association/National Heart, Lung, and Blood Institute
CI: Confidence interval
CVD: Cardiovascular disease
HR: Hazard ratio
IDF: International Diabetes Federation
HDL cholesterol: High density lipoprotein cholesterol
LDL cholesterol: Low density lipoprotein cholesterol
MetS: Metabolic syndrome
NCEP-ATP III: National Cholesterol Education Program and Adult Treatment Panel-III
RIVANA: Vascular Risk Study in Navarre
RAP: Rate advancement period
SD: Standard deviations
