# Supplementary material for: Risk for cardiovascular disease associated with metabolic syndrome and its components: a 13-year prospective study in the RIVANA cohort
Source: Cardiovasc Diabetol. 2020 Nov 22;19:195. doi: 10.1186/s12933-020-01166-6 (PMC7680587; doi:10.1186/s12933-020-01166-6)
Supplement: Supplementary file 1 — Additional file 1: Appendix S1. Diagnostic criteria for endpoints of the study. Appendix S2. Percentages of imputed information for imputed variables. Appendix S3. Causes of death, including coronary heart disease, cerebrovascular disease, other cardiovascular causes, other non-cardiovascular causes, cancer, and unknown causes, among the participants of the Rivana (Vascular Risk in Navarre) cohort (n = 3,976). Appendix S4. Kaplan-Meier estimates of the cumulative incidence of A) myocardial infarction, B) stroke, C) mortality from cardiovascular causes, and D) all-cause mortality according to the number of metabolic syndrome traits (0-1 trait, 2 traits, 3 traits, 4 traits, and 5 traits) in the Rivana cohort (n = 3,976). [file 12933_2020_1166_MOESM1_ESM.pdf]

Additional file 1

Risk for cardiovascular disease associated with metabolic syndrome and its components: a 13-year prospective study in the RIVANA cohort

María J. Guembe*, Cesar I. Fernandez-Lazaro*, Carmen Sayon-Orea, Estefanía Toledo, Conchi Moreno-Iribasand, for the RIVANA Study Investigators

* María J. Guembe and Cesar I. Fernandez-Lazaro co-first authors

Appendix S1. Diagnostic criteria for endpoints of the study.

Appendix S2. Percentages of imputed information for imputed variables.

Appendix S3. Causes of death, including coronary heart disease, cerebrovascular disease, other cardiovascular causes, other non-cardiovascular causes, cancer, and unknown causes, among the participants of the Rivana (Vascular Risk in Navarre) cohort (n=3,976).

Appendix S4. Kaplan-Meier estimates of the cumulative incidence of A) myocardial infarction, B) stroke, C) mortality from cardiovascular causes, and D) all-cause mortality according to the number of metabolic syndrome traits (0-1 trait, 2 traits, 3 traits, 4 traits, and 5 traits) in the Rivana cohort (n=3,976).

1. **Primary end point**

The primary endpoint was defined as the first occurrence of cardiovascular death, myocardial infarction, or stroke. All of these components of the primary endpoint were additionally secondary end points as defined below.

2. **Secondary endpoints**

Secondary endpoints were defined according to the International Classification of Diseases, Ninth and Tenth Revision (ICD-9 and ICD-10) and satisfying the following criteria:

A) Myocardial infarction (CIE-9: 410-411; CIE-10: I21-I22)

Criteria: One of the following criteria satisfies the diagnosis of acute myocardial infraction

- Presence of ECG (Development of pathologic Q waves in the ECG throughout the acute phase) or presence of troponins (>percentile 95) with clinical suggestion of myocardial infraction, and in the following cases:
  - Common, uncommon or poorly described symptoms, along with probable ECG (modification of the ST segment or inverted T wave during the episode) and abnormal enzymes (CK>percentile 95 or CK-MB>percentile 99) in the absence of troponins
  - Common symptoms, abnormal enzymes (CK>percentile 95 or CK-MB>percentile 99) and ischemic ECG (modification of the ST segment or inverted T wave during episode), non-codable (due to branch blockage or presence of pacemaker), or not available.
- Diagnosis from a pathological examination

B) Stroke (CIE-9: 430-438 CIE-10: I60-I69)

Acute neurological deficit lasting more than 24 hours caused by focal ischemia cerebral caused by the following syndromes: lacunar infarction, atherothrombotic infarction, infarction of cardio-embolic origin (secondary to arrhythmias, valvular diseases, or cardiopathies embolization), cerebral and subarachnoid hemorrhages. We excluded those secondary to hematological diseases (leukemias, polycythemia vera), brain metastases and head injuries. The diagnosis must be confirmed by an imaging test (CT or MRI) or confirmed by a pathological examination.

C) Mortality from cardiovascular disease

We included the following causes of death according to the International Classification of Diseases, Ninth and Tenth Revision (ICD-9 and ICD-10):

- CIE-9: 401-405, 410-414, 426-428, 429.1-429.9, 430-435, 436-438 (excluding 437.4-437.8), 443.8, 443.9, 798.1; 798.2
- CIE-10: G45-G46, I05-I09, I10-I16, I20-I25, I26-I52, I60-I69, I70-I79, I99, R00-R01

D) All-cause mortality

This end point includes all causes of death, including cardiovascular and non-cardiovascular causes. All deaths were confirmed by reviewing the National Statistics Institute of Spain.

Appendix S1. Diagnostic criteria for endpoints of the study.

Appendix S2. Percentages of imputed information for imputed variables.

Characteristic	Percentage of imputed values
At baseline	
Risk and clinical factors	
Waist circumference	0.20
BMI	0.05
Systolic BP	0.03
Diastolic BP	0.03
Total cholesterol	0.03
LDL-cholesterol	1.00
C-Reactive protein	0.03

Abbreviations: BMI, body mass index; BP, blood pressure; LDL, low density lipoprotein.

Appendix S3. Causes of death, including coronary heart disease, cerebrovascular disease, other cardiovascular causes, other non-cardiovascular causes, cancer, and unknown causes, among the participants of the Rivana (Vascular Risk in Navarre) cohort (n=3,976).

Causes of death[1]	Overall	No metabolic syndrome	Metabolic syndrome
N (frequency)	381	143 (37.5%)	238 (62.5%)
[2]Coronary heart disease	23 (6.0%)	7 (4.9%)	16 (6.7%)
[3]Cerebrovascular disease	17 (4.5%)	7 (4.9%)	10 (4.2%)
[4]Other cardiovascular causes	45 (11.8%)	14 (9.8%)	31 (13.0%)
[5]Other non-cardiovascular causes	134 (35.2%)	51 (35.7%)	83 (34.9%)
[6]Cancer	157 (41.2%)	62 (43.4%)	95 (39.9%)
Unknown causes	5 (1.3%)	2 (1.4%)	3 (1.3%)

[1]Causes of death were defined according to the International Classification of Diseases, Ninth (ICD-9) and Tenth Revision (ICD-10)
[2]Cardiovascular heart disease included the following diagnosis, CIE-9: 410-414; CIE-10: I20-I25
[3]Cerebrovascular disease included the following diagnosis, CIE-9: 430-438 (excluding 437.4-437.8); CIE-10: I60-I69
[4]Other cardiovascular causes included the following diagnosis, CIE-9: 401-405, 426-428, 429.1-429.9, 443.8, 443.9, 798.1; 798.2; CIE-10: G45-G46, I05-I09, I10-I16, I26-I52, I70-I79, I99, R001.4, R00-R01.
[5]Other non-cardiovascular causes included the rest of deceases (except unknown causes of death)
[6]Cancer included the following diagnosis, CIE-9: 140-239; CIE-10: C00-D49

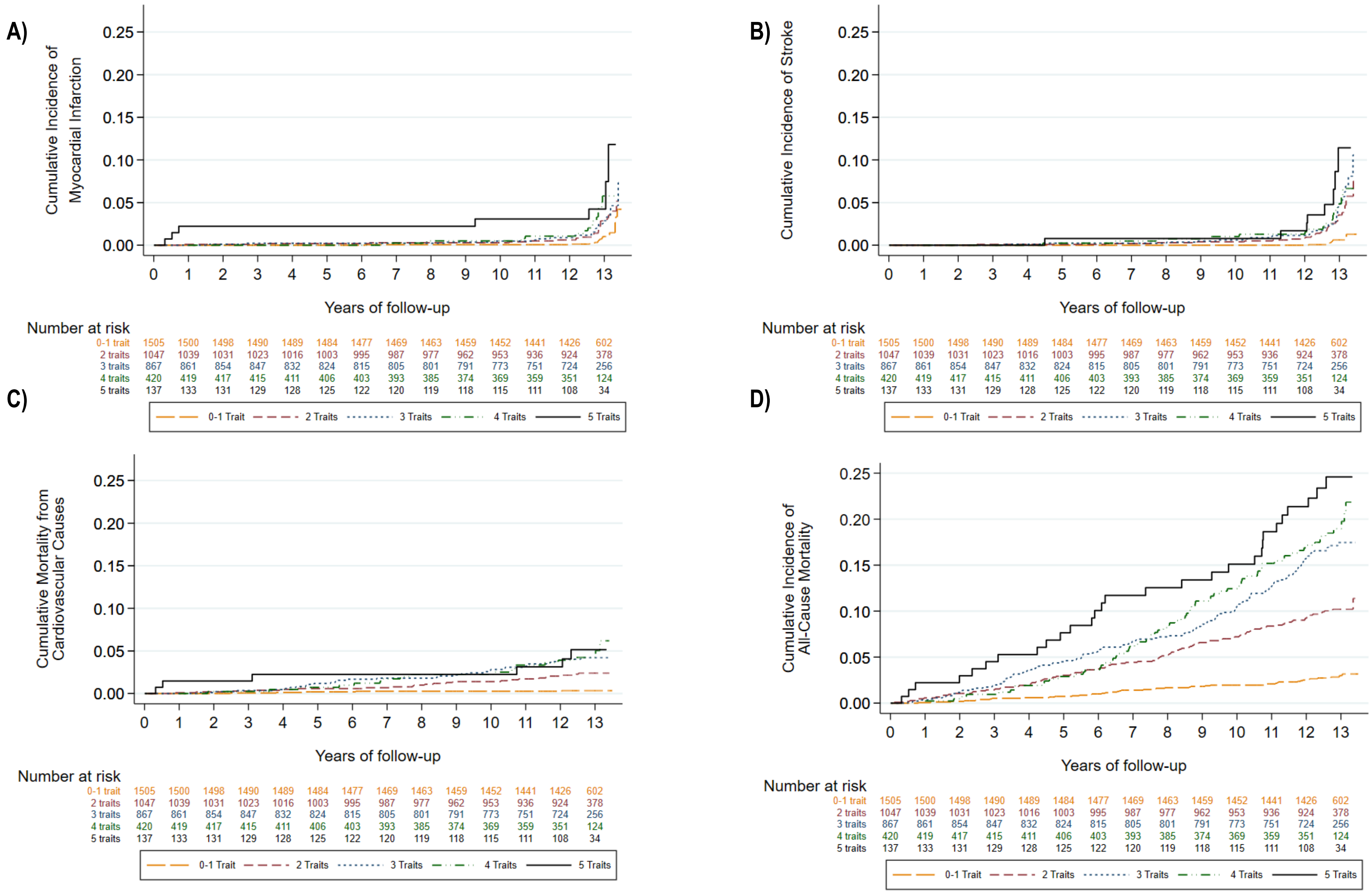

Appendix S4. Kaplan-Meier estimates of the cumulative incidence of A) myocardial infarction, B) stroke, C) mortality from cardiovascular causes, and D) all-cause mortality according to the number of metabolic syndrome traits (0-1 trait, 2 traits, 3 traits, 4 traits, and 5 traits) in the Rivana cohort (n=3,976).
